# Challenges in treating primary immune thrombocytopenia patients undergoing COVID-19 vaccination: A retrospective study

**DOI:** 10.1515/med-2024-0928

**Published:** 2024-03-23

**Authors:** Huiping Xu, Beibei Zhang, Linjun Xie

**Affiliations:** Department of Clinical Nutrition, The First Hospital of Putian City, Putian 351100, Fujian, P.R. China; Department of Hematology, The First Hospital of Putian City, Putian 351100, Fujian, P.R. China; The School of Clinical Medicine, Fujian Medical University, Fuzhou 350001, Fujian, P.R. China

**Keywords:** COVID-19, vaccination, primary immune thrombocytopenia, second-line therapy

## Abstract

**Background:**

Since the outbreak of COVID-19 in December 2019, countries around the world, including China, have been administering COVID-19 vaccines in response to the pandemic. Our center has observed that treating patients with primary immune thrombocytopenia (ITP) has become more challenging in this context.

**Methods:**

This study compared the treatment response of 25 *de novo* ITP patients who had received a COVID-19 vaccination (Group 1) with an equal number of *de novo* ITP patients randomly selected from the 2 years prior to the COVID-19 pandemic (Group 2) by using the Mann–Whitney *U* test and Fisher’s exact.

**Results:**

Patients in both groups had predominantly female gender with similar age and baseline platelet counts. However, on Day 3, the median platelets were 22 and 49 × 10^9^/L, and on Day 7, they were 74 and 159 × 10^9^/L, respectively (*P* < 0.05). Compared to Group 2, Group 1 showed a suboptimal short-term response to glucocorticoid monotherapy, with a higher proportion of patients requiring combination therapy with other drugs including intravenous immunoglobulin, thrombopoietin receptor agonists, and rituximab. After subgroup analysis, a significant difference was observed in the proportion of patients requiring second-line therapy between the two groups.

**Conclusions:**

Our study suggests that COVID-19 vaccination may lead to a lower response rate to first-line treatment in *de novo* ITP patients. Nevertheless, it is crucial to acknowledge the inherent limitations in this conclusion. Further studies are needed to confirm these findings and investigate the underlying mechanisms.

## 1 Introduction

In December 2019, a new type of coronavirus disease (COVID-19) caused by a novel coronavirus emerged in Wuhan, China, and has since become a global pandemic, infecting millions and causing numerous deaths worldwide. In response, various countries, including China, have launched mass vaccination campaigns against COVID-19. Against this backdrop, our center has noticed that the treatment of patients with primary immune thrombocytopenia (ITP) appears to be more challenging.

ITP is a bleeding disorder caused by accelerated platelet destruction and impaired megakaryocyte function. Glucocorticoids with or without intravenous immunoglobulin (IVIG) are first-line therapy options for ITP, but additional therapy may be required due to intolerance or relapse [1,2]. Although thrombocytopenia following COVID-19 vaccination has been reported, it is important to note that such cases are extremely rare and represent only a small proportion of the total number of people vaccinated globally [3–5]. Thrombosis and/or thrombocytopenia, which can occur after any COVID-19 vaccine, is associated with the activation of macrophages by the spike protein and triggers inflammation [6]. There is also evidence to suggest that the administration of COVID-19 vaccines may potentially increase the likelihood of developing ITP or exacerbate existing symptoms in individuals with pre-existing ITP [7–9].

The objective of our retrospective study is to compare the treatment response of patients with *de novo* ITP who have received the inactivated COVID-19 vaccine with a historical cohort of such patients before the COVID-19 pandemic. Although there are limitations to our retrospective study and the possibility of selection bias, our findings could have significant implications for the treatment of ITP patients who are vaccinated against COVID-19.

## 2 Methods

### 2.1 Post-COVID-19-vaccination cohort (Group 1)

We conducted a retrospective single-center analysis of all patients admitted with ITP (ICD-10 D69.3) to The First Hospital of Putian City Hematology Department from December 2020 to March 2023. Baseline clinical characteristics, treatment, and outcome were analyzed by reviewing electronic medical records. A total of 25 patients who had received the COVID-19 vaccine before admission and met the inclusion and exclusion criteria were included in Group 1.

### 2.2 Historical cohort (Group 2)

For the pre-pandemic comparison (Group 2), a cohort of 25 patients diagnosed with ITP and meeting the inclusion criteria was randomly selected from the medical records department spanning from January 2018 to December 2019 at the same hospital. Patients were assigned a unique identification number and selected using a computer-generated random number sequence. To ensure comparability with Group 1, patients in Group 2 did not receive the COVID-19 vaccine, which was not yet available during the study period.

### 2.3 Inclusion and exclusion criteria

The eligibility criteria for enrollment in both Group 1 and Group 2 entail a diagnosis of *de novo* ITP (diagnosed with ITP within the past 3 months), adult age (18 years or above), a platelet count less than 30 × 10^9^/L at admission, and complete follow-up data for a minimum of 14 days. Moreover, Group 1 patients must also satisfy the supplementary criterion of having received COVID-19 vaccination before the onset of ITP.

Patients under 18 years old, those previously treated for ITP, with other autoimmune, infectious or serious medical conditions, abnormal liver/kidney function, and pregnant/lactating women are excluded from the study to ensure study results are not confounded by pre-existing conditions or treatments.

### 2.4 Diagnostic criteria and treatment response evaluation of ITP

The diagnosis of ITP was based on the Chinese ITP Diagnosis and Treatment Guidelines [2]. Responses recorded using standard international consensus definitions (response, ≥30 × 10^9^/L and at least double baseline without bleeding; complete response, ≥100 × 10^9^/L without bleeding; failure: loss of response and/or need for additional ITP-therapy). Glucocorticoids with or without IVIG defined as first-line therapy.

While our hospital has a wealth of experience in the treatment of ITP patients and consistently adheres to both local and international guidelines [1,2], this information is provided for context and is not directly relevant to the study design or findings.

### 2.5 Statistical analyses

The descriptive statistics used in the study were median and interquartile range (IQR), mean and standard deviation, or counts with percentages.

Since the sample size was small (less than 50), the normality of the data was determined using Shapiro–Wilk test, and *P* value >0.05 was considered as normally distributed. If the data of all groups are normally distributed and have equal variances, the appropriate test to compare the difference between the two groups would be the independent samples *t*-test. If the variances are not equal, the Welch’s *t*-test should be used instead. If the data of at least one group is not normally distributed, non-parametric tests such as the Mann–Whitney *U*-test or Wilcoxon rank-sum test can be used to compare the difference between the two groups. For categorical variables, Fisher’s exact test was used.


**Ethical considerations:** To maintain patient privacy and confidentiality, all patient information was de-identified and kept secure throughout the study. Only authorized personnel had access to the data, and all electronic medical records were stored on a secure server that was password-protected. Moreover, patient data were analyzed in aggregate form to ensure that individual patients could not be identified.

## 3 Results

All patients in this study had no important organ bleeding except skin or mucosal bleeding, such as gastrointestinal bleeding, intracranial bleeding, etc., so we focused on evaluating the platelet response during treatment. Demographics are presented in Table 1.

**Table 1:** Demographics and presenting features of patients in Group 1 and Group 2

Characteristics	Group 1		Group 2	
	No. (%)	Range [IQR]	No. (%)	Range [IQR]
Female	16 (64)		17 (68)	
Age in median, years	56	26–81 [45.5–72.0]	53	20–83 [41.0–67.0]
**Platelets (×10** ^**9**^ **/L)**				
Day 1	5	0–27 [2.0–13.0]	9	1–23 [3.5–12.0]
Day 3	22	1–98 [3.5–48.5]	49	1–138 [13.0–95.5]
Day 7	74	2–304 [25.0–151.0]	159	1–520 [91.5–241.5]
Day 14	184	2–515 [40.5–337.0]	266	8–572 [129.0–353.0]
**Treatment**				
1	8 (32)		15 (60)	
2	8 (32)		7 (28)	
3	5 (20)		2 (8)	
4	4 (16)		1 (4)	

Treatment 1: glucocorticoids alone; Treatment 2: glucocorticoids in combination with IVIG; Treatment 3: sequential treatment with glucocorticoids, IVIG, and thrombopoietin receptor agonists (TPO-RA); Treatment 4: sequential treatment with glucocorticoids, IVIG, TPO-RA, and rituximab.

We can observe that both groups of patients are predominantly female with similar proportions, with median ages of 56 and 53 years, and platelets at Day 1 were 5 and 9 × 10^9^/L, respectively. In summary, the two groups of patients have similar baseline characteristics. However, only 32% of patients in Group 1 received glucocorticoids alone during hospitalization, while the proportion of such patients in Group 2 was 60%. In Group 1, a higher proportion of patients required combination therapy with other drugs including IVIG, thrombopoietin receptor agonists (TPO-RA), and even rituximab. Among those, eight patients (32%) received IVIG, five patients (20%) received both IVIG and TPO-RA, and four patients (16%) received IVIG, TPO-RA, and rituximab. In contrast, in Group 2, seven patients (28%) received IVIG, two patients (8%) received both IVIG and TPO-RA, and one patient (4%) received IVIG, TPO-RA, and rituximab.

**Figure 1:**
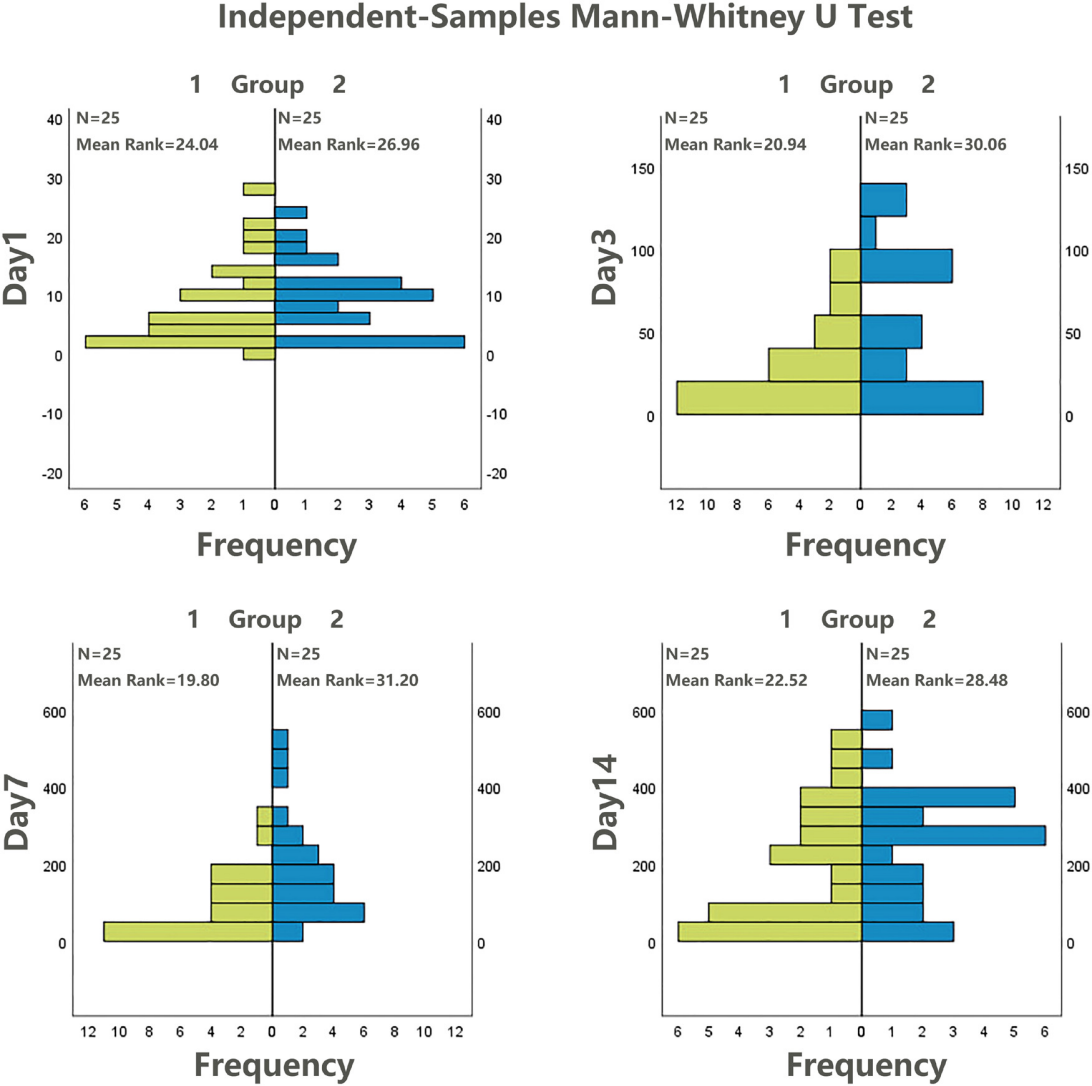
Comparison of platelets between two groups using independent-samples Mann–Whitney *U*-test.

Due to the small sample size, we assessed the normality of the data using the Shapiro–Wilk test (Table 2). As the platelet data for at least one of the groups was found to have a skewed distribution, we opted to use the Mann–Whitney *U*-test or Wilcoxon rank-sum test to compare differences between the groups for all days (Table 3 and Figure 1). On Day 1, the median platelet counts were 5 and 9 × 10^9^/L, and on Day 14, they were 184 and 266 × 10^9^/L, respectively, with no statistically significant differences observed. However, on Day 3, the median counts were 22 and 49 × 10^9^/L, and on Day 7, they were 74 and 159 × 10^9^/L, respectively, with statistically significant differences observed between the two groups (*P* < 0.05).

**Table 2:** Assessment of normality using Shapiro–Wilk test for platelet data in two groups

Shapiro–Wilk	Day 1		Day 3		Day 7		Day 14	
	1	2	1	2	1	2	1	2
Statistic	0.872	0.940	0.877	0.907	0.888	0.885	0.905	0.968
df	25	25	25	25	25	25	25	25
*P* value	0.005	0.148	0.006	0.026	0.010	0.009	0.023	0.596

1: Group 1; 2: Group 2.

**Table 3:** Comparison of platelets between two groups using Mann–Whitney *U*-test and Wilcoxon rank-sum test on different days

	Day 1	Day 3	Day 7	Day 14
Mann–Whitney *U*	276.000	198.500	170.000	238.000
Wilcoxon *W*	601.000	523.500	495.000	563.000
Z	–0.710	–2.214	–2.765	–1.446
*P* value	0.478	0.027	0.006	0.148

After conducting subgroup analysis using Fisher’s exact test (Table 4), it was observed that the gender distribution did not significantly differ between the two groups. However, a greater proportion of patients in Group 1 had platelet counts less than 30 × 10^9^/L on Day 3 than in Group 2, although this difference was not statistically significant. Furthermore, there was a significant difference between the two groups with respect to the proportion of patients having platelet counts less than 100 × 10^9^/L on Day 7, as well as requiring second-line therapy, with a higher proportion observed in Group 1 (*P* < 0.05).

**Table 4:** Subgroup analysis using Fisher’s exact test: gender distribution and platelets in two groups

	Group 1	Group 2	*P* value
Male	9	8	0.500
Female	16	17	
Platelets <30 × 10^9^/L on Day 3	15	10	0.129
Platelets ≥30 × 10^9^/L on Day 3	10	15	
Platelets <100 × 10^9^/L on Day 7	15	8	0.044
Platelets ≥100 × 10^9^/L on Day 7	10	17	
Need for second-line therapy	9	3	0.048
No need for second-line therapy	16	22	

## 4 Discussion

This study aimed to evaluate the platelet response to first-line treatment in patients with *de novo* ITP after COVID-19 vaccination. We compared the platelet counts and treatment outcomes of two groups of patients: Group 1 (*de novo* ITP patients who received COVID-19 vaccination) and Group 2 (historical *de novo* ITP patients). We found that Group 1 had a lower response rate to first-line treatment, with a higher proportion of patients requiring combination therapy and lower platelet counts on Days 3 and 7. The difference in platelet counts on Day 14 was not significant and may be related to early combination therapy improving platelet counts. The baseline characteristics of the two groups of patients were similar, indicating that the differences observed in treatment response were likely due to the effect of COVID-19 vaccination on the disease course.

In our study, 36% of patients in Group 1 required second-line therapy, consistent with Choi et al.’s report that 39% of COVID-19 vaccine-related ITP patients needed additional treatment due to poor response to first-line therapy [10]. In contrast, Saluja et al. reported that most similar cases responded well to first-line therapy [11]. The sample sizes of these three studies were all less than 100, and the differences in their results may be due to factors such as limited sample size and different types of COVID-19 vaccines administered. Further research with larger sample sizes and standardized protocols is necessary to better understand the relationship between COVID-19 vaccine administration and the treatment response of ITP.

Some studies have raised concerns about using rituximab for ITP patients during the COVID-19 pandemic due to the potential risk of infection from decreased humoral immunity after treatment [12,13]. However, thanks to the Chinese government’s strict measures for epidemic prevention and control until December 2022, we were not overly concerned about this side effect and decided to administer rituximab to four vaccinated patients with refractory ITP. The average platelets of these four patients on Day 7 and Day 14 remained low at 52 and 23.5 × 10^9^/L, respectively. Therefore, we initiated rituximab 100 mg/week for them. On Day 28, one patient was lost to follow-up, but the remaining three patients demonstrated platelet counts of 46, 123, and 237 × 10^9^/L, respectively. These results suggest that clinicians may need to be aware of the potential for poor treatment response in *de novo* ITP patients who have received COVID-19 vaccination and adjust treatment plans accordingly. Rituximab may be a useful treatment option for vaccinated ITP patients who are refractory to conventional therapies, even during the COVID-19 pandemic, with proper measures for infection control in place.

Further studies exploring the immune mechanisms involved in vaccine-related ITP may shed light on the underlying mechanisms of the observed treatment response differences. For example, it is worth investigating whether differences in antibody titers after COVID-19 vaccination may affect the response of ITP patients to first-line treatment, and whether the vaccine may induce persistent pathogenic antibody secretion in some patients through a germinal center response [14,15]. These issues warrant further basic research. In addition, some studies have suggested that COVID-19 vaccination induces a strong T-cell response, and therefore, the possibility of the involvement of cell-mediated immunity in the development of ITP in these patients also deserves exploration [16].

Although the study provides valuable insights into the treatment response of *de novo* ITP patients following COVID-19 vaccination, there are several limitations to consider. In light of constraints within our medical institution, platelet antibody testing is currently unfeasible. Due to the lack of an assessment of the concentration and specificity of antiplatelet antibodies, interference with treatment response cannot be ruled out. The lack of a prospective cohort control study design prevents us from determining whether first-line treatment can improve platelet counts in patients with poor short-term responses at Day 14 or beyond. Additionally, the local government’s promotion of COVID-19 vaccination has resulted in a lack of ITP patient data without vaccination during the same period. To address these limitations, future studies should incorporate larger sample sizes, more comprehensive assays, and prospective controlled designs, as well as data from unvaccinated ITP patients during the same period through multi-center research.

Overall, despite the limitations related to the data and study design, the study provides a useful starting point to further investigate the potential long-term effects of COVID-19 vaccination on ITP, exploring alternative treatment options for refractory cases, and conducting larger prospective controlled studies to confirm the observed treatment response differences.

## 5 Conclusion

Our study suggests that *de novo* ITP patients who received COVID-19 vaccination had a lower response rate to first-line treatment compared to those prior to the pandemic. A higher proportion of vaccinated patients required combination therapy and had lower platelet counts on Day 3 and Day 7. This highlights the importance of close monitoring and tailored treatment for vaccinated *de novo* ITP patients. However, our study still has certain limitations, including the absence of platelet antibody testing and a lack of a prospective cohort control study, which precludes the exclusion of potential confounding factors, including the possible interference of different vaccines with treatment outcomes. Larger studies are needed to confirm our findings and explore the potential impact of COVID-19 vaccination on ITP development and progression.
